# Supplementary material for: Ultrafast long-range spin-funneling in solution-processed Ruddlesden–Popper halide perovskites
Source: Nat Commun. 2019 Aug 1;10:3456. doi: 10.1038/s41467-019-11251-4 (PMC6671992; doi:10.1038/s41467-019-11251-4)
Supplement: Supplementary file 1 — Supplementary Info [file 41467_2019_11251_MOESM1_ESM.pdf]

Supplementary Information

Ultrafast long-range spin funneling in solution-processed Ruddlesden-Popper halide perovskites

D. Giovanni, *et al.*

Supplementary Note 1:

Transient Absorption Setup

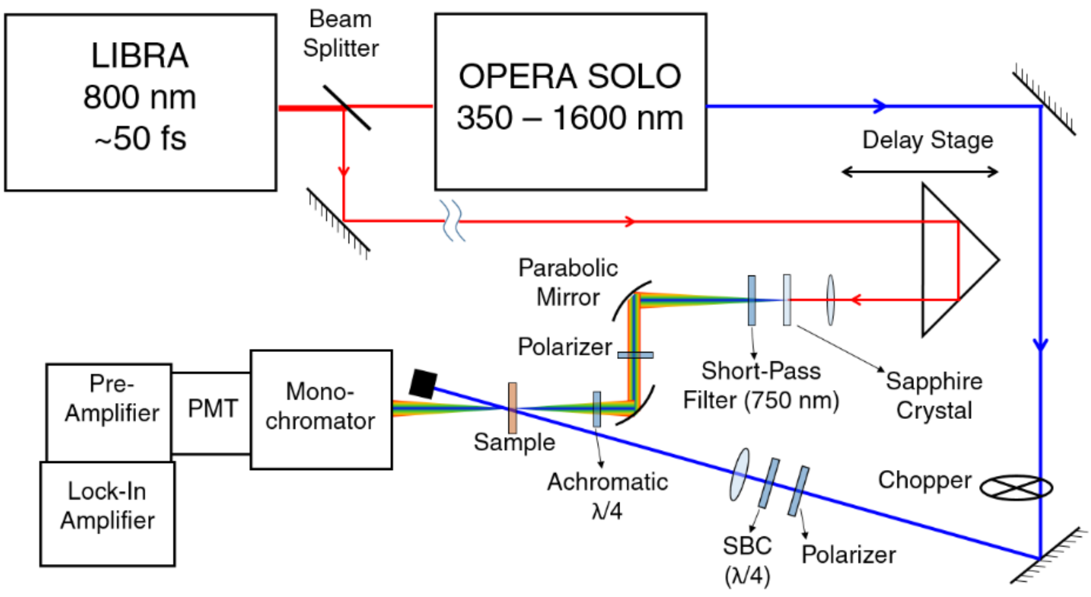

Supplementary Figure 1 | Transient Absorption (TA) spectroscopy setup. The schematic of our home-built TA setup used in this work for circularly pump and probe measurements. The circular polarizations for the pump and probe are generated using a Solei Babinet Compensator (SBC) and an achromatic quarter-waveplate, respectively.

The transient absorption setup is a home-built system powered by the 800 nm Coherent Inc. *LIBRA*^{TM} Ti:Sapphire laser with ~50 fs pulse and 1 kHz repetition rate. The output is split into two beams: one to the optical parametric amplifier (*Coherent OPeRa SOLO*^{TM}) to generate tunable photon energy (pump) and another beam to a delay stage (probe). The 800 nm probe beam from the delay stage is then focused to a sapphire crystal for white light generation (1.5 eV – 2.8 eV), as shown in Supplementary Figure 1. The measurements were performed in transmission mode. Transmitted probe was collected and sent to monochromator and photo multiplier tube (PMT), before being sent into a pre-amplifier and a lock-in amplifier. The pump

beam is mechanically chopped frequency of 83 Hz. To generate circularly polarized beam, linear polarizers were used for both pump and probe, before a Solei Babinet Compensator (SBC, for pump) and an achromatic quarter-waveplate ($\lambda/4$, for probe) were added at ±45° with respect to the polarizers' axis. Excitation fluence control in this experiment was performed by using variable density filters. Chirp-correction for different probe energies was also implemented on-site.

Supplementary Note 2:

Perovskites optical spin selection rules

The spin-orbit coupling (SOC) modifies the band edge of perovskites from s-like valence band and p-like conduction band to $|m_J\rangle$ = ±1/2 angular momentum (J) states[1-4]. The band structure calculation of lead halide perovskites at R-point reveal the orbital contributions to the valence and conduction bands (VB and CB) around the bandgap. The VB originates mainly from Pb(6s) I(5p) orbital contributions, while the CB comes mainly from Pb(6p) orbitals. The presence of spin-orbit coupling (SOC) splits the CB (L = 1) into lower J = 1/2 and upper J = 3/2 states, while leaving the VB (L = 0) unaffected. Focusing on the band edge dynamics (i.e., VB maximum with J = S = 1/2 and bottom-most CB with J = 1/2), we can consider both bands as doubly degenerate (m_J = ±½). Therefore, the contribution to the spin-state can be obtained by calculating the Clebsh-Gordan (CG) coefficients, given the strong SOC of perovskites:

$$|L,S,J,m_j\rangle = \sum_{m_l}\sum_{m_s}\langle L,S,m_l,m_s|L,S,J,m_s\rangle|L,S,m_l,m_s\rangle \quad (1)$$

where S = 1/2, L = 1 for CB and L = 0 for VB. The CG coefficient is zero for $m_J = m_L + m_S$, while addition tables will give the non-zero components. Resulting states for CB and VB are:

$$\left|1,\frac{1}{2},\frac{1}{2},+\frac{1}{2}\right\rangle_{CB} = -\sqrt{\frac{1}{3}}\left|1,\frac{1}{2},0,+\frac{1}{2}\right\rangle + \sqrt{\frac{2}{3}}\left|1,\frac{1}{2},+1,-\frac{1}{2}\right\rangle \quad (2a)$$

$$\left|1,\frac{1}{2},\frac{1}{2},-\frac{1}{2}\right\rangle_{CB} = -\sqrt{\frac{2}{3}}\left|1,\frac{1}{2},-1,+\frac{1}{2}\right\rangle + \sqrt{\frac{1}{3}}\left|1,\frac{1}{2},0,-\frac{1}{2}\right\rangle \quad (2b)$$

$$\left|0,\frac{1}{2},\frac{1}{2},+\frac{1}{2}\right\rangle_{VB} = \left|0,\frac{1}{2},0,+\frac{1}{2}\right\rangle \quad (2c)$$

$$\left|0,\frac{1}{2},\frac{1}{2},-\frac{1}{2}\right\rangle_{VB} = \left|0,\frac{1}{2},0,-\frac{1}{2}\right\rangle \quad (2d)$$

Taking the modulus square of the respective coefficients, the ‘J-up’ ($m_J = +½$) state consists of 33% spin-up and 67% spin-down electrons, while the ‘J-down’ ($m_J = –½$) state consists of 67% spin-up and 33% spin-down electrons. With total angular momentum $J = ½$, conservation of angular momentum dictates that the absorption of $\sigma^{\pm}$ photon will result in the change of azimuthal quantum number (m_J) by ±1 (i.e., $\Delta m_J = \pm 1$). Hence, optical excitation of J- (spin-) polarized carriers/excitons with 100% polarization using circularly polarized light can be achieved. Fig. 1c (Main Text) illustrates the optical selection rules of circularly polarized light in lead halide perovskites. in the case of 2D perovskites, some relaxation of these selection rules can be expected when the light polarization is out-of-plane with respect to the quantum-well plane[2]. This maybe the reason of the imperfect (< 100%) spin-polarization observed in our experiments.

Supplementary Note 3:

Slow charge transfer process in RPP thin films.

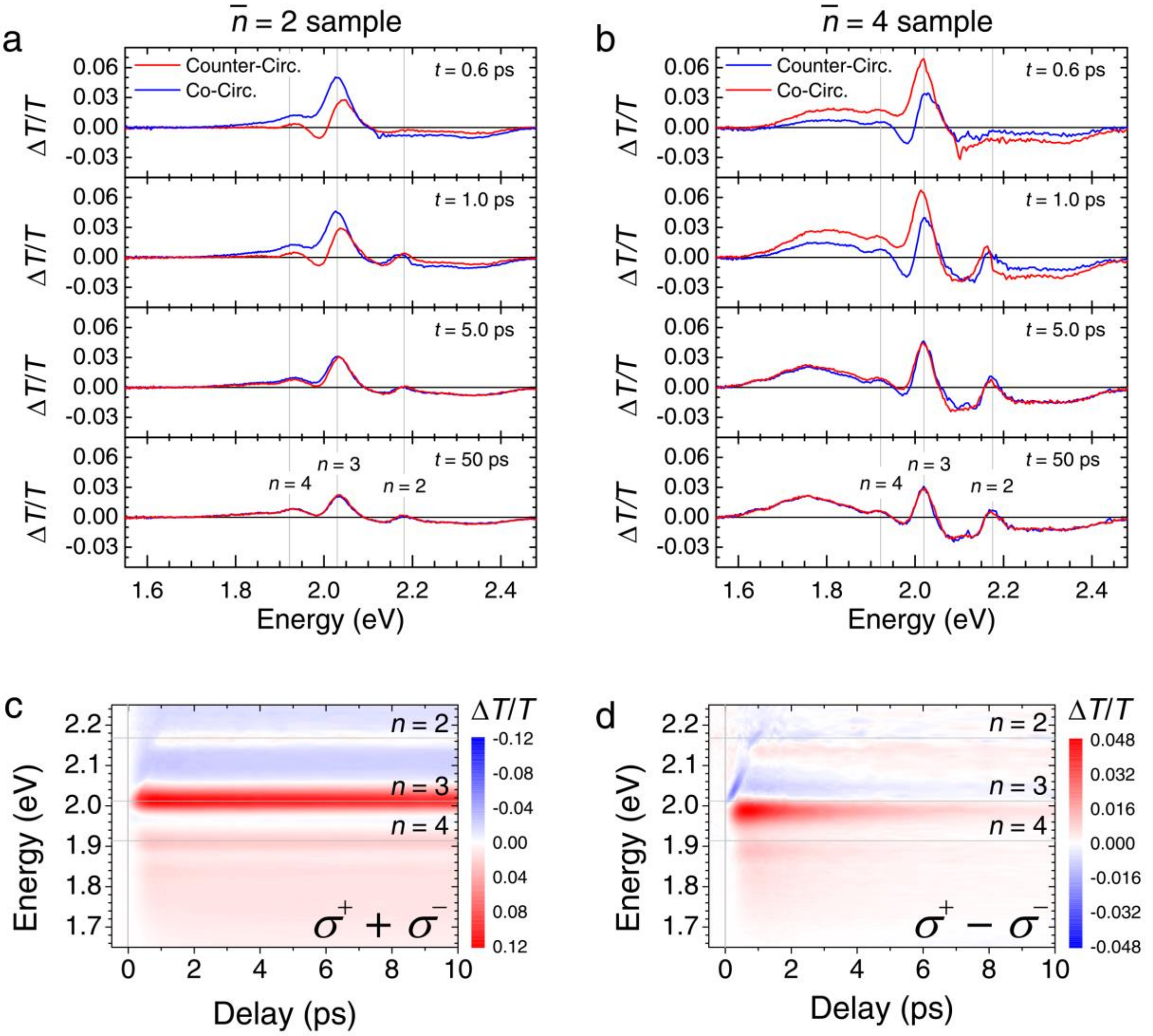

Supplementary Figure 2 | Transient absorption (TA) spectra of Ruddlesden-Popper perovskite at various time delay. The TA spectra of our **(a)** $\bar{n}$ = 2 sample and **(b)** $\bar{n}$ = 4 sample photoexcited by 2.07 eV pump. The n = 2 PB peak at 2.17 eV were formed at later time delay, signifying the presence of a slower charge transfer process. The contour $\Delta T/T$ vs time vs $\hbar\omega$ plot for our $\bar{n}$ = 4 sample of **(c)** $\sigma^+ + \sigma^-$ probe signal and **(d)** $\sigma^+ - \sigma^-$ probe signal.

Apart from initial ultrafast exciton funneling, a slower charge transfer process is also observed in both of our RPP thin film samples. This is evident from the emergence of PB n = 2 peak at 2.17 eV for time delay ≥ 1 ps (Supplementary Figure 2), regardless of our lower pump

energy (2.07 eV) compared to the n = 2 band gap. Since energy transfer process will only allow transfer from initial to lower energy levels, this observation could be unanimously assigned to a charge transfer process, which occurs in the timescale of ~1 to 5 ps. Unlike the ultrafast energy funneling process, no spin-polarization is observed after the charge transfer process.

To better understand the picture, the contour plot of $\Delta T/T$ as a function of time and probe energy is presented in Supplementary Figure 2c-d. Here, Supplementary Figure 2c shows the sum of dynamics of the co- and counter-circular polarizations, which represents the total exciton population (i.e., indiscriminate of the spin states). The slightly delayed positive signal at the n = 2 phase resonance after t = 1 ps implies a slower charge transfer process. Meanwhile, the net spin polarization signal in Supplementary Figure 2d shows a zero net-spin after n = 2 state-filling; except the sharp negative spike at about t = 1 ps, which arises from the slow rise dynamics of the broad PA band. Other features on the net spin signal in Supplementary Figure 2d to be highlighted are the negative spike on the higher energy side of n = 3 resonance due to complex interplay at the isobestic point of the n = 3 excitonic peak; and the large positive peak on the low energy side of n = 3 resonance due to the different signs of σ^+ and σ^- probe signal at this intermediate states (IS) region. Furthermore, the delayed rise of the net signal in both the low and high energy side of n = 3 peak (which is resonantly pumped) implies: spin funneling to the lower bandgap phases, and that the PA signal at the higher energy side comes from further absorption of lower bandgap phases to higher energy states.

Supplementary Note 4:

Observation of intermediate state (IS) by 2.25 eV pump

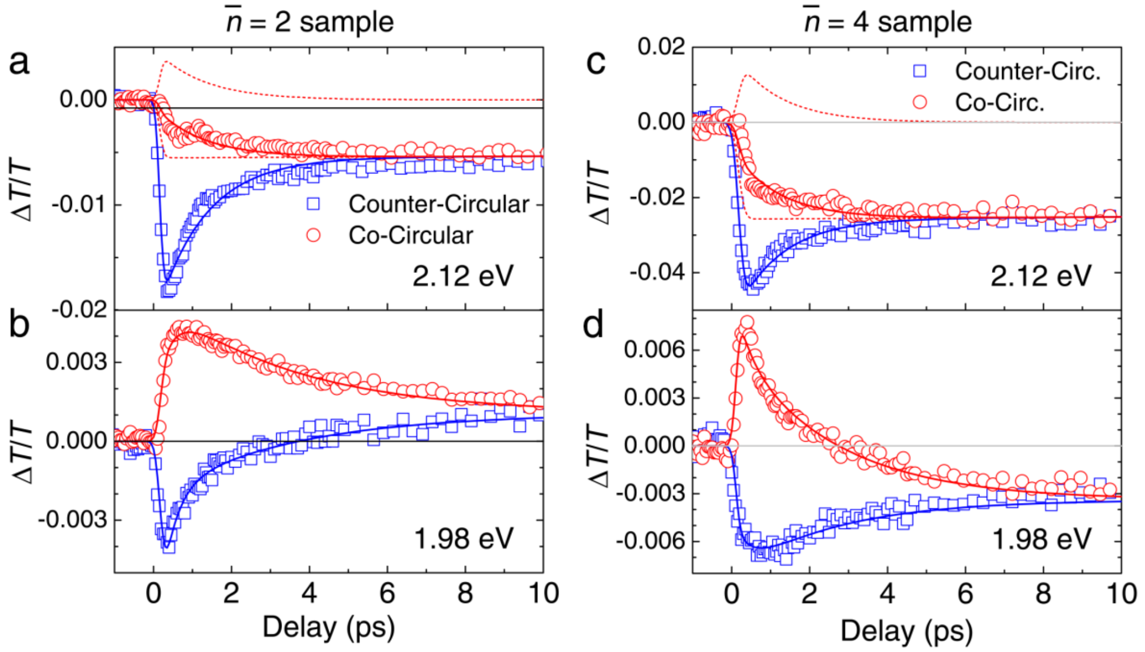

Supplementary Figure 3 | Kinetics of Ruddlesden-Popper perovskites probed at intermediate state (IS) level. The kinetics are photoexcited by 2.25 eV pump and probed at 2.12 eV (top, in between n = 2 and n =3 phases) and 1.98 eV (bottom, in between n = 3 and n = 4 phases) for **(a, b)** $\bar{n}$ = 2 sample and **(c, d)** $\bar{n}$ = 4 sample. The dashed lines in the top figures are the deconvolved contribution from photobleaching (PB) and photoinduced absorption (PA) components to the co-circular kinetics.

Supplementary Figure 3 shows the kinetics for $\bar{n}$ = 2 and $\bar{n}$ = 4 samples photoexcited by 2.25 eV pump, probed at intermediate states (IS) $\hbar\omega$ = 2.12 eV (between of n = 2 and n = 3 phases) and $\hbar\omega$ = 1.98 eV (between of n = 3 and n = 4 phases). The observed kinetics here are similar with 2.07 eV pumped kinetics (i.e., Fig. 3c-d in Main Text), where co- and counter-circular pump-probe polarization exhibit different signs of TA signal [positive (PB) and negative (PA), respectively] but with shared lifetimes. Such observation strongly suggests the presence of IS in between the two adjacent phases (see Main Text). While it is not so obvious for 2.12 eV probe, the co-circular polarization also contains a short-lived PB contribution, which is buried

in the stronger long-lived negative spin-independent signal; as compared to counter-circular polarization with full PA signal (i.e., both short-lived and long-lived). The short-lived contributions (PB and PA for co- and counter-circular, respectively) share an identical lifetime.

Supplementary Note 5:

Modelling the trap-limited spin-funneling in RPP

Based on our findings of intermediate state (IS) traps, we developed a trap-limited energy diffusion model that describes the spin-polarization trend from spin-funneling observed in our results. Herein, we assume a continuous distribution of energies. The initial photoexcitation by the pulsed laser will create a population of excitons concentrated at 2.07 eV. Due to interphase traps, these photoexcited excitons will then diffuse non-linearly towards the low-bandgap phases (i.e., the diffusion coefficient will be density dependent). The following equation can phenomenologically model such a process:

$$D(\rho) = D_0(1 - \exp\{\rho/\rho_T\}) \tag{3}$$

Where D is the diffusion coefficient; ρ is the carrier density; D_0 is a constant; and ρ_T is the interphase trap-density. This equation describes an increase in the diffusion coefficient (excitons diffuse more efficiently) when the traps are filled. The time dynamics of spin-up ($\rho_\uparrow$) and spin-down ($\rho_\downarrow$) populations at RPP phase with bandgap energy E can be described by the following set of differential equations:

$$\begin{aligned} \frac{\partial}{\partial t}\rho_\uparrow(E,t) &= \frac{\partial}{\partial E}\left[D(\rho_\uparrow)\frac{\partial}{\partial E}\rho_\uparrow\right] - \left(\frac{\rho_\uparrow - \rho_\downarrow}{\tau_s}\right) \\ &\quad + \left(\frac{1+P_0}{2}\right)e^{-(E-E_0)^2/2\Delta^2}\, e^{-(t-2\tau_0)^2/\tau_0{}^2} \end{aligned} \tag{4a}$$

$$\begin{aligned} \frac{\partial}{\partial t}\rho_\downarrow(E,t) &= \frac{\partial}{\partial E}\left[D(\rho_\downarrow)\frac{\partial}{\partial E}\rho_\downarrow\right] + \left(\frac{\rho_\uparrow - \rho_\downarrow}{\tau_s}\right) \\ &\quad + \left(\frac{1-P_0}{2}\right)e^{-(E-E_0)^2/2\Delta^2}\, e^{-(t-2\tau_0)^2/\tau_0{}^2}. \end{aligned} \tag{4b}$$

Here, the first term describes the energy diffusion/funneling, the second term describes the spin relaxation, and the third term describes the initial photoexcitation of the spin-polarized excitons; τ_s is the spin relaxation lifetime, P_0 is the initial spin-polarization, E_0 is the laser energy, Δ is the laser spectral bandwidth, and τ_o is the laser pulse width. Hard-wall boundary

condition is applied at 1.64 eV (3D band-edge) and 2.05 eV (few tens of meV above n = 3 phase).

From the model, we extracted on the spin-polarization at 0.6 ps. The contribution to the population from 'thermalization within itself' $\rho_{\uparrow}^{T}(E)$ and $\rho_{\downarrow}^{T}(E)$ is given by:

$$\rho_{\uparrow}^{T}(E) = \left[\eta e^{-(E-E_C)^2/\Sigma^2}\right]\left[1 - e^{-(E-E_0)^2/2\Delta^2}\right]\left[\frac{1}{2} + \frac{1}{2}e^{-(E-E_0)^2/\alpha^2}\right] \qquad (5a)$$

$$\rho_{\downarrow}^{T}(E) = \left[\eta e^{-(E-E_C)^2/\Sigma^2}\right]\left[1 - e^{-(E-E_0)^2/2\Delta^2}\right]\left[\frac{1}{2} - \frac{1}{2}e^{-(E-E_0)^2/\alpha^2}\right]. \qquad (5b)$$

The first-factor describes the distribution of RPP phases in our samples, which is assumed to be Gaussian, centered at E_C with a spread of Σ. The second-factor describes the correction for the double-counting of photoexcitation from the funneling. Lastly, the third-factor describes the decay of polarization due to momentum-scattering in thermalization process. The result of our model is shown in Fig. 4c.

Supplementary Note 6:

Effect of energy shift on the calculation of spin-polarization

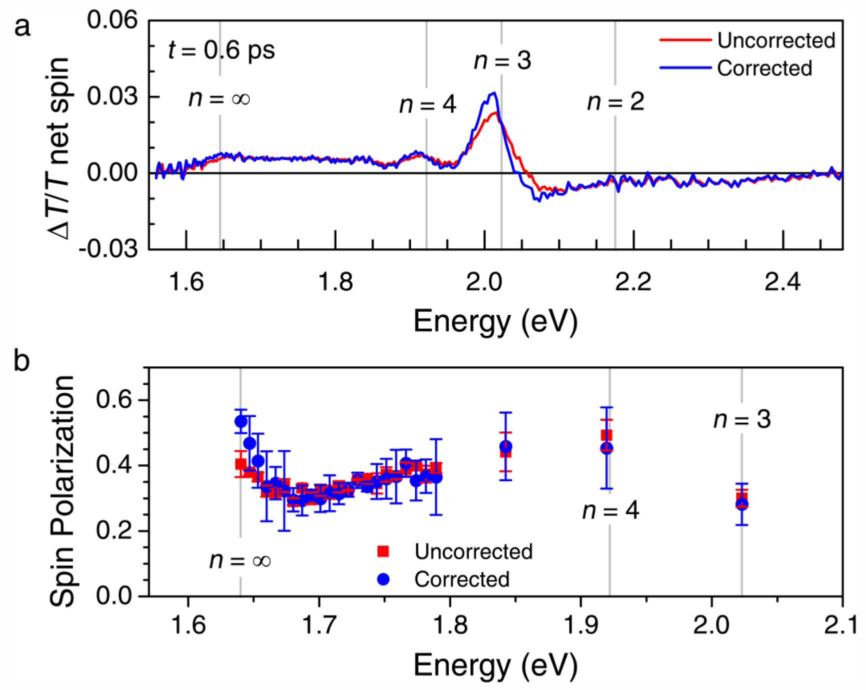

Supplementary Figure 4 | Pseudo-spin correction due to energy shift. Comparison between uncorrected (red) and corrected (blue) signal of (**a**) net-spin and (**b**) spin-polarization of the graded 2D/3D sample. The net-spin signal is obtained from co-circular subtracted with counter-circular TA signal. The error-bars are obtained from the standard deviation of the spin-polarization measured within the probe spectral bandwidth of 3 nm.

A small apparent shift (~6 meV) was observed in the TA spectra between the counter- and co-circular peaks (Fig. 3a-b and Fig. 4b in Main Text), which may cause artefact in the spin-polarization calculation. Herein, this contribution of artefact is estimated. The net-spin signal $S(E)$ [i.e., $S = \Delta T/T_{\text{(co-circular)}} - \Delta T/T_{\text{(counter-circular)}}$] has two contributions: from the real difference in spin-population S_P, and from the energy shift S_S:

$$S = S_P + S_S. \quad (6)$$

The change $S_S(E)$ from a signal spectrum $y(E)$ due to a shift of ΔE is given by a differential shape of the spectrum:

$$S_{\mathrm{S}} = -\frac{\partial y}{\partial E}\Delta E. \tag{7}$$

Therefore, we have a correction for the ‘pseudo-polarization’ induced by the shift:

$$S_{\mathrm{P}}(E) = S(E) + \Delta E \frac{\partial}{\partial E}\left(\frac{\Delta T}{T}\right)_{\mathrm{Co-circular}}. \tag{8}$$

Here, the $\Delta T/T_{\text{(co-circular)}}$ spectrum is assumed to arise dominantly from the exciton population. The net-spin signal with correction $S_{\mathrm{P}}(E)$ and without correction $S(E)$ are shown in Supplementary Figure 4a. The calculated spin-polarization with and without correction are shown in Supplementary Figure 4b. A huge increase of uncertainty from the spin polarization propagates from the error in the spectral derivative. Importantly, the correction does not affect both the spin-polarization trend and our previous interpretation of the paper.

Supplementary Note 7

Derivation of exciton spin relaxation dynamics modelling

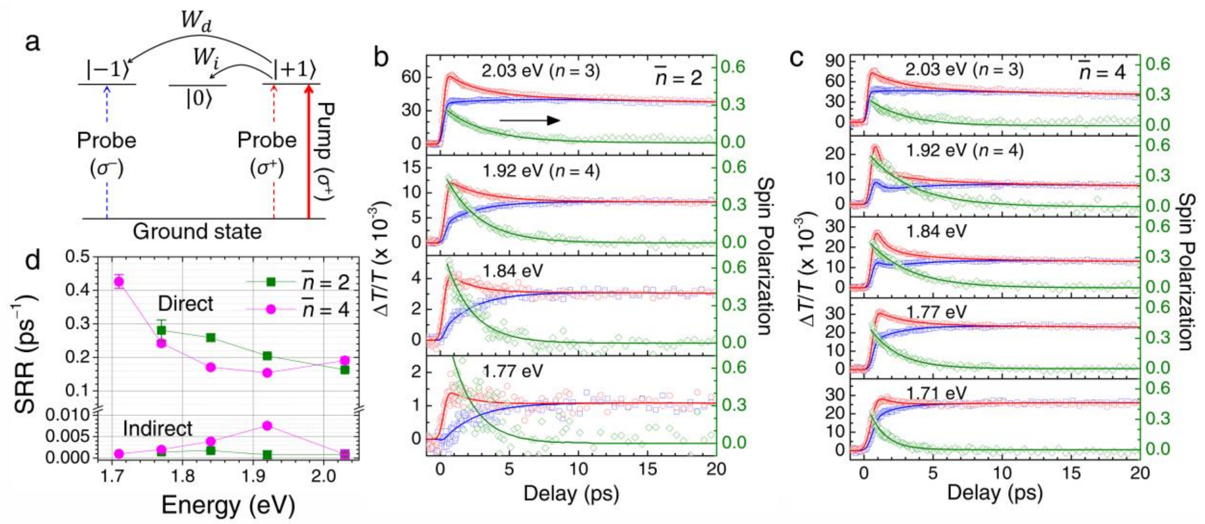

Supplementary Figure 5 | Exciton spin relaxation dynamics in Ruddlesden-Popper perovskites (RPP). (a) Exciton spin-states in RPP: two bright exciton states $|+1\rangle$ and $|-1\rangle$ and a dark exciton state $|0\rangle$. After photoexcitation by σ^+ pump to $|+1\rangle$ exciton state, the spin polarization relaxes to $|0\rangle$ and $|-1\rangle$ states with indirect (W_i) and direct (W_d) spin-flip rate, respectively. **(b, c)** $\Delta T/T$ signal of co-circular (red) and counter-circular (blue) pump-probe polarization for **(b)** $\bar{n}$ = 2 and **(c)** $\bar{n}$ = 4 samples. The spin-polarizations (green) are plotted in the right-Y axis. The experimental data is well-fitted with Supplementary Equation 19. **(d)** The extracted spin relaxation rate (SRR) of RPP at different probe energies.

After photoexcitation by the σ^+ pump and the thermalization/funneling process, the exciton spin-polarization P at the band edge/detection level is defined as follows:

$$P(t) = \frac{f_{+1} - f_{-1}}{f_{+1} + f_{-1}} = \frac{G(t)}{F(t)}. \tag{9}$$

Here, $f_{\pm 1}$ is the exciton population in the $|\pm 1\rangle$ exciton spin-state. For mathematical convenience, we defined $F(t) \equiv f_{+1} + f_{-1}$ and $G(t) \equiv f_{+1} - f_{-1}$. Based on the model in Supplementary Figure 5a, the kinetics of each exciton spin state can be written as:

$$\frac{d}{dt}\begin{pmatrix} f_{+1} \\ f_0 \\ f_{-1} \end{pmatrix} = \begin{pmatrix} -(W_d+W_i) & W_i & W_d \\ W_i & -2W_i & W_i \\ W_d & W_i & -(W_d+W_i) \end{pmatrix}\begin{pmatrix} f_{+1} \\ f_0 \\ f_{-1} \end{pmatrix}. \tag{10}$$

The W_d and W_i are the direct and indirect spin-flip rate, respectively[5,6]. The direct spin-flip means the electron and hole simultaneously flip their spin from $|\pm 1\rangle$ state to $|\mp 1\rangle$ state; while indirect spin-flip means only one of either the electron/hole in an exciton flips its spin from $|\pm 1\rangle$ state to $|0\rangle$ state. Here, $|0\rangle$ state is a dark state, i.e., optically inaccessible. Using the definition of $F(t)$ and $G(t)$, the equation can be re-written in terms of F and G as follows:

$$\frac{dF}{dt} = -W_i F + 2W_i f_0 \tag{11a}$$

$$\frac{dG}{dt} = -(2W_d + W_i)G \tag{11b}$$

$$\frac{df_0}{dt} = W_i F - 2W_i f_0 \tag{11c}$$

Substituting these equations to eliminate $f_0(t)$, we obtained the second order differential equation of $F(t)$:

$$\frac{d^2F}{dt^2} + 3W_i\frac{dF}{dt} = 0. \tag{12}$$

The general solution for such differential equation is given by:

$$F(t) = Ae^{\gamma_1 t} + Be^{\gamma_2 t}. \tag{13}$$

Herein, $\gamma_{1,2}$ is the root of quadratic equation $x^2 + 3W_i x = 0$, which is given by $\gamma_1 = -3W_i$ and $\gamma_2 = 0$. The general solution for $F(t)$ is therefore given by:

$$F(t) = Ae^{-3W_i t} + B. \tag{14}$$

The constant A and B can be solved by applying the boundary conditions:

$$F(t=0) = C_0, \tag{15a}$$

$$f_0(t \to \infty) = \frac{1}{2}F(t \to \infty). \tag{15b}$$

Here, C_0 is the initial photoexcited population of exciton. Hence, we obtained:

$$F(t) = \frac{C_0}{3}(e^{-3W_i t} + 2). \tag{16}$$

Meanwhile:

$$G(t) = C_1 e^{-(2W_d + W_i)t}, \tag{17}$$

where the boundary condition $G(t = 0) = C_1$ has been applied. The initial spin polarization therefore is given by $P_0 = C_1/C_0$. The populations of the spin-states $f_{\pm 1}$, are related to $F(t)$ and $G(t)$ by:

$$f_{\pm 1}(t) = \frac{1}{2}[F(t) \pm G(t)]. \tag{18}$$

The $\sigma^{\pm}$ probe readings, which are proportional to the populations of the spin-states, are therefore given by:

$$\sigma^{\pm}(t) = \frac{1}{2}\left[\frac{1}{3}e^{-3W_i t} + \frac{2}{3} \pm P_0 e^{-(2W_d + W_i)t}\right] X(t). \tag{19}$$

Here, $X(t) = \sum_i A_i \exp(-t/\tau_i)\,[1 + \mathrm{erf}(t/r - r/2\tau_i)]/2$ is the multi-exponential envelop function with lifetime τ_i, which takes care of the spin-independent total dynamics of the excitons. The kinetics of PB peaks/band for $\bar{n}$ = 2 and $\bar{n}$ = 4 samples at different $\hbar\omega$ are shown in Supplementary Figure 5b-c. The observed dynamics are well-fitted by this model, implying the accuracy of our description. Similar to the case of $CH_3NH_3PbI_3$ (or MAPI)[3], sub 10-ps spin relaxation is observed in all of the regions probed. It implies the significance of SOC to the spin relaxation process in perovskites, regardless of its structural dimensionality. The direct and indirect spin relaxation rates (SRR) were extracted from the fitting and presented in Supplementary Figure 5d. Our results show that for all RPP phases, the indirect exciton spin relaxation to the dark states (W_i ~ 0.001 ps^{-1}) play a negligible role as compared to the direct-flip mechanism (W_d ~ 0.02 ps^{-1}), i.e., $W_i << W_d$. Interestingly, a general trend of increasing direct SRR increases with decreasing band gap energy (or increasing phase number n) is

observed in RPP thin films, except for n = 1, where the SRR is the highest (~5 ps^{-1}, see next section). Further investigation is required to understand this phenomenon.

Supplementary Note 8:

Spin relaxation of *n* = 1 phase

The net spin signals (i.e., $\sigma^{+} - \sigma^{-}$ probe) for σ^{+} excitation at various fluences for $n = 1$ $(C_6H_5C_2H_4NH_3)_2PbI_4$ are shown in Supplementary Figure 6. The single exponential fitting yields fluence independent lifetime of 0.20 ± 0.02 ps. This lifetime corresponds to a spin relaxation rate of 5.0 ± 0.5 ps^{-1}, which is much higher than other RPP $n > 1$ phases.

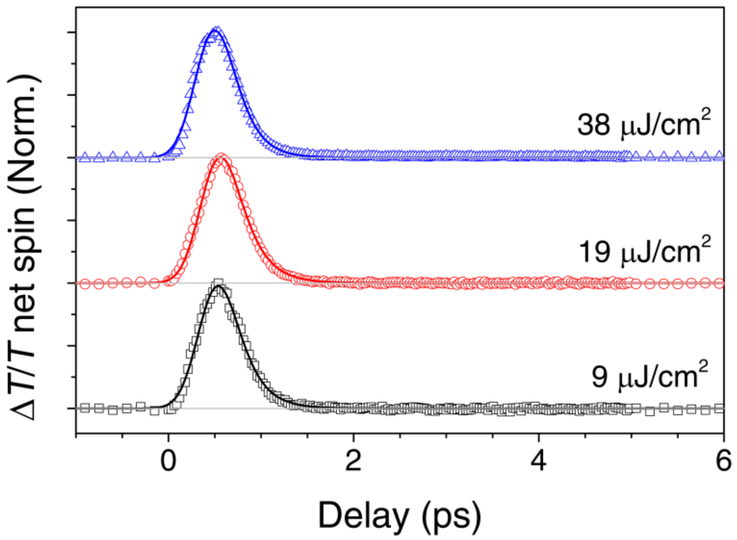

Supplementary Figure 6 | Net spin signal of $(C_6H_5C_2H_4NH_3)_2PbI_4$ *n* = 1 thin film. The observed kinetics are resonantly pumped and probed at 2.41 eV. The fitting yields fluence independent lifetime of 0.20 ± 0.02 ps, which correspond to a spin-relaxation rate of 5.0 ± 0.5 ps^{-1}.

Supplementary Note 9:

Thickness measurement of 2D/3D layered samples

Supplementary Figure 7 shows the 2D/3D layered sample measured by Atomic Force Microscopy (AFM) over tens of μm scanning range. A scratch was made over the sample as the base height reference. Measurements over several positions on the sample were performed.

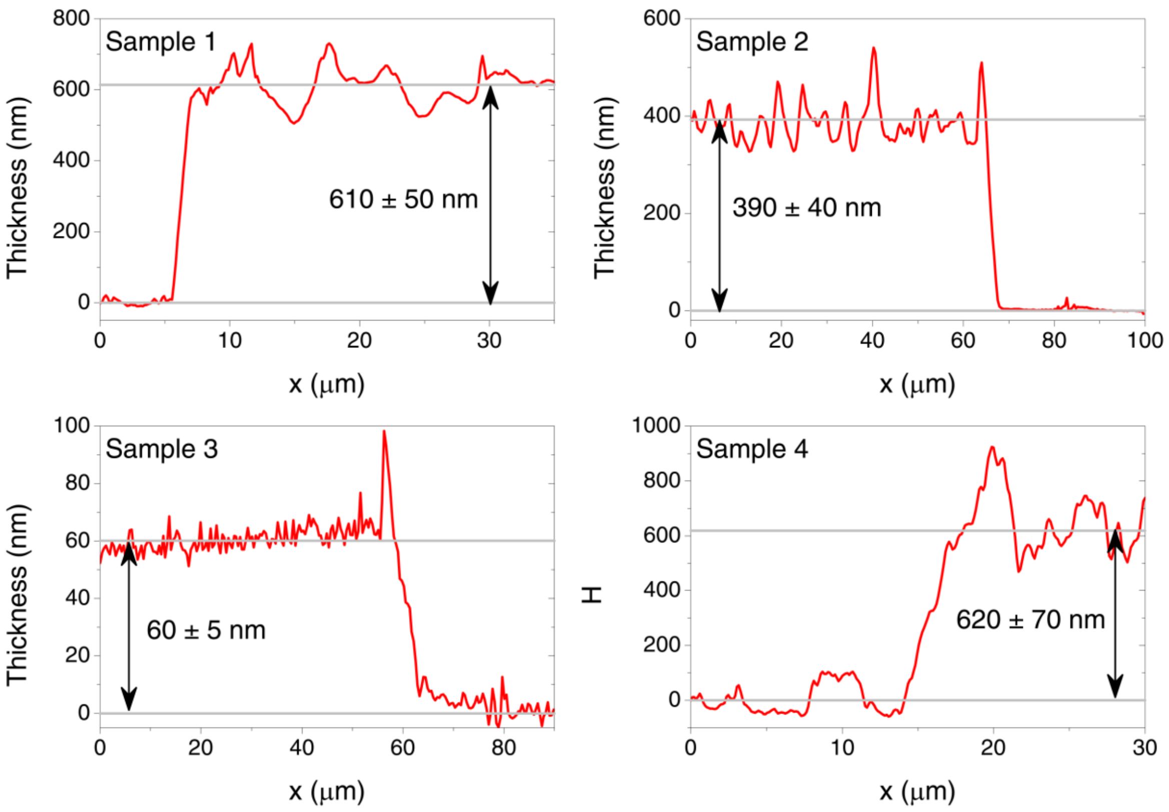

Supplementary Figure 7 | AFM measurement on 2D/3D layered samples. AFM thickness measurements of several 2D/3D graded samples.

Supplementary Note 10:

Photoluminescence (PL) and reflectance spectrum of 2D/3D graded sample

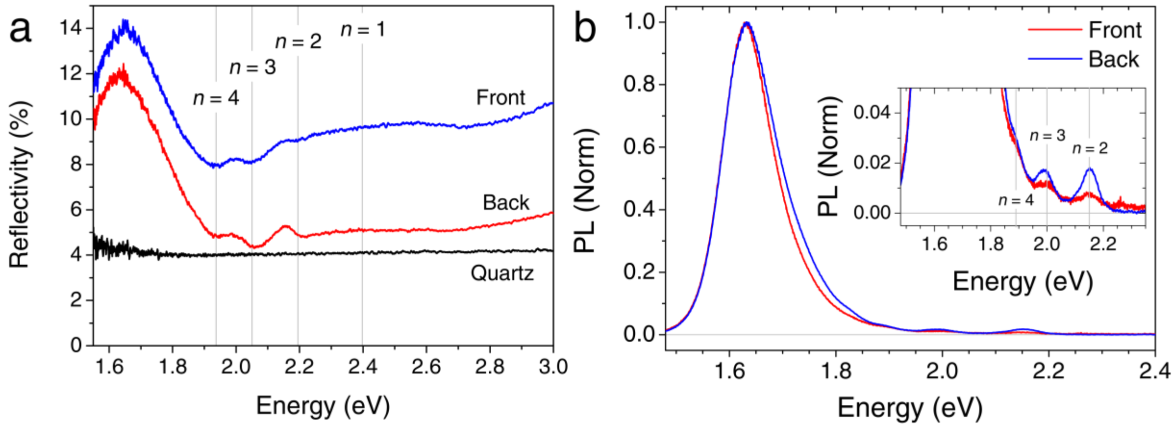

Supplementary Figure 8 | Reflectivity and PL spectra of our 2D/3D layered sample. (a) Reflectivity spectra, taken at normal incidence from the back (red) and front (blue). Measurement on quartz is also provided as reference. **(b)** PL spectra photoexcited from the front and back sides of the sample. Inset: PL intensity shows more dominant emission from low-*n* phases at back excitation.

The reflectivity spectra taken at normal incidence of our 2D/3D graded samples are shown in Supplementary Figure 8a. The reflectivity from the front of the sample shows a less prominent 2D perovskite peaks as compared to the back reflectivity. For further confirmation, PL spectra (at glancing incident excitation ~80°) from two different faces of the sample are shown in Supplementary Figure 8b. The PL spectrum with back-side excitation (i.e., substrate/film interface) shows clear signatures of $n < 4$ RPP peaks; while from the front-side excitation (film/air interface), such peaks are not apparent. A broader main PL peak towards high energy emission is also observed by back-side excitation, which implies higher contribution from the low *n* phases. All these observations verify the 2D/3D layered structure of our sample (see Fig. 4a in Main Text).

Supplementary Note 11:

Effect of magnetic field on spin lifetime

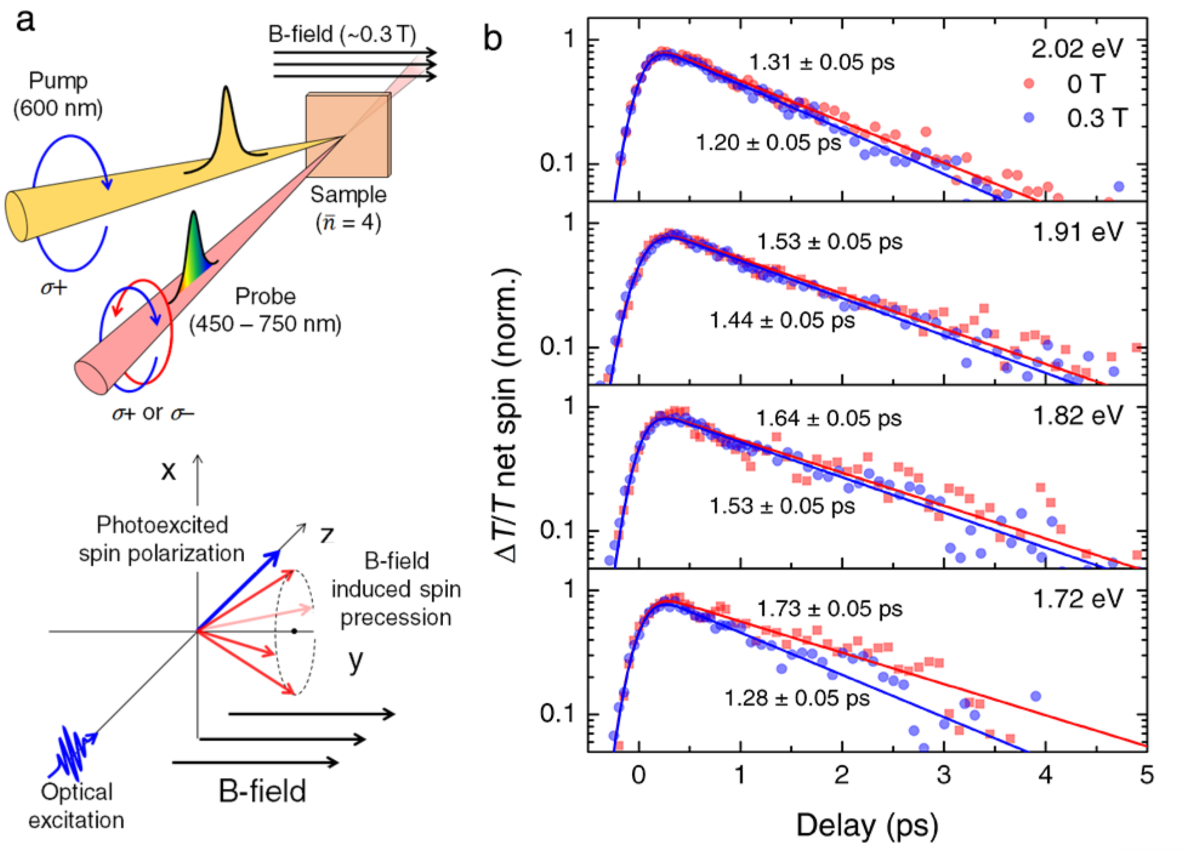

Supplementary Figure 9 | Modified Hanle effect measurements on perovskites. (a) The circularly-polarized pump-probe geometry for measurement of the spin lifetime, in the presence of perpendicular magnetic field. The spin-precession along *y*-axis induced by the B-field will shorten the lifetime of the detected spin-polarization at *z*-direction. **(b)** The exciton spin lifetime measured at different probe energies for the $\bar{n}$ = 4 RP perovskite sample. Small yet consistent shortenings of the spin-lifetimes are observed in the presence of a B-field.

Herein, we performed the measurements of exciton spin relaxation time with and without magnetic field perpendicular to the sample surface on our $\bar{n}$ = 4 sample (Supplementary Figure 9). Similar to the case of optical Hanle effect measurement, the presence of the magnetic field perpendicular (at *y*-direction) to the initial photogenerated spin-polarization (along *z*-direction)

will trigger the spin to precess along the y-axis, hence lower the spin-polarization (or shorten the spin lifetime) detected optically at the z-direction. In our case, the actual optical Hanle effect measurement is not possible, since the spin lifetime is much shorter than the radiative recombination lifetime in perovskites at room temperature.

To the best of our capabilities, we performed such measurements at room temperature (~300 K) with maximum magnetic field (B-field) of ~0.3 T. Based on the perovskites Lamour frequency of $\omega_L/2\pi$ ~ 10 GHz at B-field of 300 mT[7]; and with given the spin lifetime τ_s of ~2 ps at room temperature, we expect a shortening of spin lifetime due to B-field by factor of $(\omega_L\tau_s)^2/[1 + (\omega_L\tau_s)^2]$ ~ 2%. As expected, we observed a small yet consistent shortening of the measured spin lifetimes – Supplementary Figure 9b. Unfortunately, measurements at low temperature (~10 K) with higher magnetic field, which will yield more contrasting results, is beyond our capability.

Supplementary References:

1. K. Tanaka *et al.* Comparative study on the excitons in lead-halide-based perovskite-type crystals $CH_3NH_3PbBr_3$ $CH_3NH_3PbI_3$, *Solid State Commun.***,** 127, 619 (2003).
2. T. Kenichiro *et al.* Electronic and Excitonic Structures of Inorganic–Organic Perovskite-Type Quantum-Well Crystal $(C_4H_9NH_3)_2PbBr_4$, *Jpn. J. Appl. Phys.***,** 44, 5923 (2005).
3. D. Giovanni *et al.* Highly Spin-Polarized Carrier Dynamics and Ultralarge Photoinduced Magnetization in $CH_3NH_3PbI_3$ Perovskite Thin Films, *Nano Lett.***,** 15, 1553 (2015).
4. D. Giovanni *et al.* Tunable room-temperature spin-selective optical Stark effect in solution-processed layered halide perovskites, *Sci. Adv.***,** 2, 1600477 (2016).
5. M. Z. Maialle, E. A. de Andrada e Silva & L. J. Sham. Exciton spin dynamics in quantum wells, *Phys. Rev. B***,** 47, 15776 (1993).
6. T. Amand & X. Marie. in *Spin Physics in Semiconductors* (ed Michel I. Dyakonov) 55 (Springer Berlin Heidelberg, 2008).
7. P. Odenthal *et al.* Spin-polarized exciton quantum beating in hybrid organic-inorganic perovskites, *Nat. Phys.***,** 13, 894 (2017).
